# Psychological morbidities among Nepalese migrant workers to Gulf and Malaysia

**DOI:** 10.1371/journal.pone.0267784

**Published:** 2023-11-08

**Authors:** Abha Sharma, Renuka Adhikari, Enjila Parajuli, Manisha Buda, Jyotika Raut, Ena Gautam, Bibhav Adhikari

**Affiliations:** 1 Faculty of Nursing, Mahidol University, Salaya, Thailand; 2 Janamaitri Foundation Institute of Health Sciences, Lalitpur, Nepal; 3 Central Department of Home Science, Tribhuvan University, Kirtipur, Nepal; University of Alabama at Birmingham, UNITED STATES

## Abstract

**Background:**

One of the important aftereffects of rapid global development is international mobility, which has placed the health of migrant workers as a key public health issue. A less-developed country, Nepal, with political instability and a significant lack of employment, could not remain untouched by this phenomenon of migration. Our goal was to identify and determine the predictors of anxiety, depression, and psychological wellbeing among Nepalese migrant workers in Gulf countries (United Arab Emirates, Saudi Arabia, Qatar, Oman, Kuwait, Bahrain) and Malaysia.

**Methods:**

A descriptive cross-sectional study was used to collect information from 502 Nepalese migrant workers in the arrival section of Tribhuvan International Airport from May to June 2019 using purposive sampling. Workers with a minimum work experience of 6 months and above were included in the study. A structured questionnaire with socio-demographic items was used along with the Beck Depression Inventory (BDI), Beck Anxiety Inventory (BAI) and WHO (five) wellbeing scale for measuring the subjective psychological wellbeing and screening for depression.

**Results:**

The mean age of the respondents was 32.97 years. Majority (41.8%) of the respondents had work experience in Qatar and 63.7% had work experience of 1–5 years. The results suggested that 14.4% had mild to severe depression while 4.4% had a moderate level of anxiety. The WHO5 wellbeing index score suggested that 14.1% of the respondents had a score below 13, which is suggestive of poor psychological wellbeing. Further, the country of work (p = 0.043), sleeping hours (p = 0.001), occupation (p = 0.044), working hours (p = 0.000), water intake (p = 0.010) and anxiety level (p = 0.000) were found to be significantly associated with depression score. Similarly, sleeping hours (p = 0.022), occupation (p = 0.016), working hours (p = 0.000), water intake (p = 0.010), and anxiety level (0.000) were significantly associated with the WHO5 wellbeing score.

**Conclusions:**

Nepalese migrant workers in the Gulf countries (United Arab Emirates, Saudi Arabia, Qatar, Oman, Kuwait, Bahrain) and Malaysia bear an important burden of psychological morbidities. This highlights the need to prioritize the migrant worker’s mental health by Nepal as well as Gulf countries and Malaysia.

## Introduction

Rapid global development has increased international mobility. Over last four decades, international migration has doubled from 82 million in 1970 to 200 million in 2005 [1]. At present, around one billion population is mobile [2]. The International Organization for Migration (IOM) defines a migrant as a person who moves away from their place of usual residence, whether within a country or across an international border, temporarily or permanently, and for a variety of reasons [3].

Further, it divides migration into four categories: labor migration, forced migration or displacement, human trafficking and modern slavery, and environmental migration. In 2017, 59% only were labor migrants, which in numbers was 164 million [3].

Labor migration, as the name suggests, is for employment in a foreign territory. Labor migrants are those who seek paid work in a different country than theirs. According to United Nations estimates, more than two-thirds of the approximately 175 million migrants worldwide represent migrant workers and their families [4]. This has placed migrant’s health at risk, and thus, migrant health has become a key public health issue worldwide.

International Labour Organization (ILO) states that one of the major destinations for migrant workers from around the globe have been the Arab states [5]. Migrant workers have made a significant contribution to the development of these countries, while they are also the major source of remittances for their own. Data shows that, in 2019, there were 35 million international migrant workers in the Gulf Cooperation Council (GCC) countries, along with Jordan and Lebanon, and the majority of them were from Asia [5].

Within Asia too, Nepal is one developing country, with elongated history of political instability and unemployment, and hence, not new to this phenomenon. Each Nepali household now at least one member involved in foreign employment, and thus, this has become a primary source of income for the same [6]. The trend of leaving the country for temporary jobs internationally is on rise since the early 2000s, and the major destinations are countries like Qatar, Saudi Arabia, UAE, Kuwait and Malaysia. When there were only 3,605 labour approvals issued by the Department of Foreign Employment (DOFE) in 1993/94, by 2018/19, the number increased by sixty times (236,208) [6].

With the shifting demographics in the country, the socio-economic aspect is changing drastically [7, 8]. A quarter of Nepal’s GDP depends on the remittances now, which has increased from 2.5 to 8.8 billion dollars in a span of eight years [6]. In 2016 alone, the remittance received was 31.3% of then Nepal’s GDP [9].

While there is tremendous improvement in the economy, with the increased migration there came slew of health problems too, and these have affected both the countries of origin and destination. It has been found out that various socio-cultural factors such as discrimination, language and cultural barriers, legal positions and socio-economic status manifest health issues in the migrants [10]. In the context of Asian immigrants in the Middle East, migrants from poorer groups were found to be at high risk of mental illness [11].

Further, migrant workers in Gulf countries have to work in harsh conditions [12], and majority of South Asian migrant workers there working as construction workers, domestic helpers, cleaners, and drivers, have lower pay, longer working hours, and physically and mentally hazardous work and home settings [13, 14]. Females are even more vulnerable as they are at high risk of being victims of physical, sexual and verbal abuse [11, 15]. The lifestyle pattern of these Nepalese migrant workers to Gulf and Malaysia depict that they have unhealthy lifestyle pattern which posit risk for their mental and physical health which has been reported elsewhere [16].

Talking about mental health, a study conducted by the Canadian Task Force concluded that even though migration did not lead to mental illness directly, the accumulation of other risk factors has been found to compromise mental health [17]. Only few other studies have been conducted which have shown the links between psychiatric disorders and migration, despite such issues are in increasing trend [18–20].

Nonetheless, there have been some efforts made by various host nations to address these problems among the migrants, such as Qatar banning outdoors work for midday hours in summer, imposing a minimum wage rule and working with specific governments of nations providing labour migrants such as those of Nepal and Ethiopia to mitigate more issues. However, the employers and sponsors, who have been benefitting from exploiting work of the labourers have been strongly resisting these measures by the respective administrations [21–23].

This shows that there is a need of more studies to be done to understand the health issues faced by the labor migrants in depth in alignment with the cultural, linguistic and family structural differences in the destination and host countries, which also include other details such as issues of worship, diet, dress code, etc. [12].

Specifically, to understand the mental health perspective of the migrants, there needs to be greater understanding of their living and working conditions with the socio-cultural factors. This is even more pronounced when reports on the labour migration reflect the number of rescue requests made by the workers to their respective embassies in their destination countries, which echo their distress. With very limited studies exploring mental health of this portion of the population, there seems to be a great need for such studies to take place.

Thus, this study was conducted with the intention to identify and determine the predictors of anxiety, depression, and psychological wellbeing among Nepalese migrant workers in Gulf countries and Malaysia.

## Materials and methods

### Ethical considerations

The research was conducted only after the approval of the concerned authorities (University Grant Commission, Tribhuvan International Airport, and Janamaitri Foundation Institute of Health Sciences). Ethical approval was obtained from the Nepal Health Research Council (NHRC) (NHRC reference number is 2913). A written consent was obtained from each participant prior to the data collection. There was no discrimination based on caste, religion, socioeconomic status etc.

Participants were assured that confidentiality would be maintained by not disclosing the names of the participants. Participants were assured that the collected data would be used only for research purposes. Social and cultural values were respected. All the data will be kept in a cupboard safely by the researcher until 6 months after the completion of the research.

### Research design and setting

We conducted a descriptive cross-sectional study at the arrival section of Tribhuvan International Airport (TIA), which is the only international airport in Nepal. The data collection center was installed within the premises of the airport from May through June 2019.

### Study population and sample size

Nepali migrant workers arriving at the Tribhuvan International Airport from Gulf countries (Bahrain, Kuwait, Oman, Qatar, Saudi Arabia, United Arab Emirates) and Malaysia were the study population.

Nepalese citizens working in any occupation and rank in the mentioned countries, residing there for a minimum of 6 months, arriving at the Tribhuvan International Airport during the period of data collection, having no prior diagnosis of mental illness and participating voluntarily were included in the study.

We calculated the sample size at the level of 95% significance level, permissible error of 5%, and it was 502.

A purposive sampling technique was used to obtain the required sample. It was collected during the course of one month.

### Study variables and instruments

A structured questionnaire was developed incorporating socio-demographic variables, the Beck Depression Inventory (BDI), the Beck Anxiety Inventory (BAI) and WHO Well Being Index (5).

Socio-demographic variables observed were age, education, religion, address, country of work, occupation, and other work-related variables such as work experience, working hours per day, work temperature, room sharing, sleep hours, and water intake.

The Beck Depression Inventory (BDI) and Beck Anxiety Inventory (BAI) are 21-item scales used to assess depression and anxiety symptoms over the prior two weeks. Items are scored 0–3 with an instrument range of 0 to 62. Both scales have been validated for use in Nepal. The area under the curve (AUC), which captures the number of correctly classified people in this case for moderate depression or anxiety, was 0.919 (95% CI 0.878–0.960) for the BDI and 0.847 (95% CI 0.789–0.906) for the BAI; internal reliability was also high: BDI Cronbach’s α = 0.90 and BAI α = 0.90. Based on clinical validation of the BDI in Nepal, a score of 20 or higher suggests moderate depression with the need for mental health intervention (sensitivity = 0.73, specificity = 0.91) [24, 25].

The WHO (Five) Wellbeing Index is a short, self-reported measure of current mental wellbeing. It assesses subjective psychological wellbeing. It was first introduced in its present form in 1998 by the WHO Regional Office in Europe as a part of the DEPCARE project on wellbeing measures in primary health care [26]. It is suitable for people aged 9 and above. It has five statements which must be rated on a scale (0 indicating at no time and 5 indicating all of the time) in relation to the past two weeks. The total score ranges from 0–25 and is multiplied by 4 for final scoring, where 0 represents the worst imaginable wellbeing and 100 represents the best [26]. The WHO 5 wellbeing index has been found to have adequate validity for screening depression and in measuring outcomes in clinical trials [27].

The WHO 5 wellbeing index was translated into the Nepali language. Double and back translation of the instrument were done to acquire semantic equivalence between the source language and the target language [28]. The Cronbach alpha of the translated version was 0.842.

Though BAI, BDI and WHO5 wellbeing index had to be answered referring to the previous two weeks from the time of the survey, socio-demographic and other lifestyle questionnaires were not time-bound.

### Data collection procedure

The purpose of the study was explained to the participants, and they were recruited based on the inclusion criteria. An informed written consent was obtained from each of the participants, and once the consent was signed, the data was collected. Participants with a higher level of education preferred to complete the questionnaire by themselves, while others preferred answering questions face-to-face with a member of the research team. Even though some of the respondents filled out the questionnaire by themselves, they worked on the socio-demographic part only, and for the rest of the questionnaire (BDI, BAI and WHO5), all of them consulted the research team.

The research team included six people (Abha Sharma, Renuka Adhikari, Enjila Parajuli, Manisha Buda, Jyotika Raut, and Ena Gautam), who worked in two shifts (3 in each) at the arrival section. All the questionnaires were in Nepali and interviews were also conducted in Nepali language. Among the six members, two are lecturers in nursing school (1^st^ and 2^nd^ authors), two are clinical registered nurse and two were final-year graduate level nursing students. Before the data collection, 2 training sessions were organized for the research team. And each shift included one nursing lecturer, a clinical nurse, and a final year student. All the respondents had either been interviewed or consulted the research team while completing the data set, so there was no missing data.

The study has included both migrant workers who have returned home permanently as well as those who are visiting family on work leave. But the migrant workers who returned after 3 months of departure for being disqualified from the job that they were supposed to do were excluded.

### Data management and analysis

Independent variables considered in the Analysis of Variance (ANOVA) included socio-demographic variables including age (15–25 years, 26–35 years, 36–45 years, 46–55 years and 56–65 years), education (primary, secondary, higher secondary), religion (Hindu, Muslim, Buddhist, Christian and Kirat), ecological address in Nepal (Terai, Hill and Mountain), country of work (GCC countries and Malaysia), occupation, work experience (1–5 years, 6–10 years, 11–15 years, 16–20 years and 21–25 years) and other selected variables; working hours per day (up to 7 hours, 8–12 hours, 13–17 hours and 18–22 hours), work temperature (20–30 degrees, 31–40 degress, 41–50 degress and 51–60 degree celsius), room sharing (1–5 people, 6–10 people, 11–15 people, 16–20 people and 21–25 people), sleep hours per day (2–4 hours, 5–8 hours and 9–14 hours), water intake per day (1–3 liters, 4–6 liters, 7–9 liters and 10–12 liters). These categories were developed to reflect the minimum and maximum values of the included variables. During the analysis categories were not merged and altered despite some of the categories had very small frequencies. As these variables represent their sociodemographic and living conditions, they were considered to be included for identifying predictors of wellbeing and depression among Nepalese migrant workers.

After collecting the data, it was checked for completeness, organized, coded, and entered into SPSS version 20. Descriptive statistics like frequency, percentage, mean, and standard deviation, as well as inferential statistics, ANOVA was used. Considerations were made with 95% confidence interval, 5% permissible error and P value less than or equal to 0.05. All population had a common variance, and they were independent of each other.

## Results

Table 1 shows that 43.2% respondents are from the age group of 26–35 years. The mean age was 32.97 (SD = 7.62). The majority (93%) of the respondents were male. Most of the respondents (81%) were married. Nearly half (47%) of the respondents had completed their secondary level education, and only 17.3% of the respondents had completed higher secondary level education. The religion status reveals that the majority of respondents (84.3%) were Hindu, whereas the Gulf countries are Muslim, indicating that Nepalese migrants face different cultural and religious contexts as they adjust. It also shows that more than half (55.60%) of the respondents were from Terai and 7.40% were from the mountains. These regions show the temperature variations in Nepal, which affect the temperature adoptability. Regarding work experience, it shows that nearly half (41.8%) of the respondents had work experience in Qatar, followed by Saudi Arabia (21.3%). In considering occupation status, 25.9% were involved in construction work, followed by driving (12.9%). More than half (56.8%) of the respondents worked in the indoor area, while 12.9% worked in both indoor and outdoor areas and 63.7% of the respondents had 1–5 years of work experience. The majority, 86.9% worked for 8–12 hours per day, and 27.5% of respondents had to work in temperatures between 41 and 50 degrees Celsius, while 6.8% had to work in temperature above 50 degrees Celsius. Regarding room sharing, 31.5% of respondents shared a room with 6–10 people. In all, a majority of respondents (88.6%) slept for 5–8 hours, while 4.8% slept for less than 4 hours.

**Table 1:** Sociodemographic characteristics of respondents N = 502.

Characteristics	Category	Frequency	Percent
Age group (Years)	15–25	100	19.9
	26–35	217	43.2
	36–45	156	31.1
	46–55	27	5.4
	56–65	2	.4
	Mean Age: 32.97, SD: 7.62		
Sex	Male	466	93
	Female	36	7
Marital Status	Married	410	81.7
	Unmarried	92	18.3
Education	Primary level	179	35.7
	Secondary Level	236	47
	Higher Secondary Level	87	17.3
Religion	Hindu	423	84.3
	Muslim	39	7.8
	Buddhist	24	4.8
	Christian	11	2.2
	Kirat	5	1
Ecological Address in Nepal	Terai	279	55.60
	Hill	186	37.10
	Mountain	37	7.40
Country of work	Qatar	210	41.8
	Saudi Arabia	107	21.3
	UAE	106	21.1
	Kuwait	35	7.0
	Oman	14	2.8
	Bahrain	5	1.0
	Malaysia	25	5
Occupation	Office work	39	7.8
	Maintenance	56	11.2
	Aviation	7	1.4
	Factory workers	24	4.8
	Food industry	59	11.8
	Sales	33	6.6
	Construction	130	25.9
	Security	35	7.0
	Driving	65	12.9
	Garment	12	2.4
	home maid/housekeeping	42	8.4
Area of work	Indoor	285	56.8
	Outdoor	152	30.3
	Both	65	12.9
Work Experience (In years)	1–5 years	320	63.7
	6–10 years	118	23.5
	11–15 years	50	10.0
	16–20 years	12	2.4
	21–25 years	2	.4
Working Hours per day	3–7	12	2.4
	8–12	436	86.9
	13–17	35	7.0
	18–22	19	3.8
Work Temperature	20–30	259	51.6
	31–40	71	14.1
	41–50	138	27.5
	51–60	34	6.8
Room sharing	1–5 people	331	65.9
	6–10 people	158	31.5
	11–15 people	7	1.4
	16–20 people	4	.8
	21–25 people	2	.4
Sleep hours	Less than 4 hours	24	4.8
	5–8 hour	445	88.6
	9–14 hours	33	6.6
Water intake per day	1–3 liters	256	51
	4–6 liters	209	41.6
	7–9 liters	30	6
	10–12 liters	7	1.40

Table 2 presents the anxiety level of the respondents. It shows that the majority (95.2%) of the respondents had a low level of anxiety, whereas 4.4% of the respondents had a moderate level of anxiety.

**Table 2:** Anxiety level of the respondents (Beck Anxiety Inventory).

Anxiety Level	Frequency	Percent
Low	478	95.2
Moderate	22	4.4
Severe	2	0.4
Total	502	100.0

Table 3 presents depression level of the respondents. It depicts that 85.7% of the respondents had no depression, whereas 8% of the respondents had mild mood disturbance, 2.6% had borderline, 3.2% had moderate and 0.6% of the respondents with severe depression.

**Table 3:** Depression level of the respondents (Beck Depression Inventory).

Level of Depression	Frequency	Percent
Normal	430	85.7
mild mood disturbance	40	8.0
Borderline	13	2.6
Moderate	16	3.2
Severe	3	0.6
Total	502	100.0

Table 4 presents wellbeing of respondents. It shows that 14.1% had a score on the WHO5 wellbeing index of below13, which indicates poor psychological wellbeing. The mean score was 18.229, with SD of 4.71.

**Table 4:** WHO5 wellbeing index level of the respondents.

WHO5 Wellbeing score	Frequency	Percent
0–13	71	14.1
14–25	431	85.9
Total	502	100.0

Mean score = 18.22, SD = 4.71, Minimum = 0, Maximum = 25

Table 5 shows that country of work (F = 2.087, p = 0.043), sleeping hours (F = 6.963, p = 0.001), occupation (F = 1.890, p = 0.044), working hours (F = 6.630, p = 0.000), water intake (F = 4.776, p = 0.003), and anxiety level (F = 98.187, p = 0.000) were significantly associated with depression score as measured by BDI.

**Table 5:** Analysis of variance comparisons between depression score with study variables.

Country	Mean	Std. Deviation	F	Sig.
Qatar	4.4571	6.14113	2.087	.043
Saudi Arabia	3.5607	5.94975		
UAE	3.4231	6.16708		
Kuwait	6.4857	7.37791		
Oman	8.2857	9.98790		
Bahrain	2.4000	2.30217		
Malaysia	5.4800	7.05998		
Abu Dhabi	3.0000	2.82843		
**Sleep hours**				
Less than 4 hours	8.9583	8.58957	6.963	.001
5–8 hours	4.1416	6.19177		
9–14 hours	3.4242	6.22008		
**Occupation**				
Office work	4.5897	7.82613	1.890	.044
Maintenance	4.3929	6.46298		
Aviation	2.0000	2.88675		
Factory workers	5.4167	6.39916		
Food industry	3.7288	6.33227		
Sales	3.4545	5.39149		
Construction	4.3231	6.17976		
Security	2.2857	3.97471		
Driving	3.7077	6.07691		
Garment	5.3333	8.29385		
Home maid /housekeeping	7.6429	7.48599		
**Working hours**				
3–7 hours	5.4167	9.53899	6.630	.000
8–12 hours	4.0138	6.07991		
13–17 hours	4.4571	6.86141		
18–22 hours	10.5263	7.61846		
**Water intake**				
1–3 Liter	5.2461	7.08146	4.776	.003
4–6 Liter	3.6651	5.72887		
7–9 Liter	1.8667	3.19194		
10–12 Liter	.8571	2.26779		
**BAI level**				
Low	3.5732	5.39682	98.187	.000
Moderate	18.5455	5.83689		
Severe	27.5000	10.60660		

Table 6 shows the Tukey post hoc test which revealed that the depression score as per BDI was significantly lower among respondents with 5–8 hours of sleep and 9–14 hours of sleep per day compared to those with less than 4 hours of sleep. Regarding occupation, depression scores were significantly higher among respondents in the home maid/ housekeeping group than those working for security, and nearly significant (P = 0.067) than those involved in driving. Respondents working 18–22 hours had significantly higher scores for depression than those working 8–12 hours and 13–17 hours. Also, respondents drinking 1–3 liters of water were found to have a significantly higher score on depression than those drinking 4–6 liters and 7–9 liters of water. Regarding anxiety level as per BAI, respondents with moderate and severe anxiety had significantly higher scores for depression compared to those with low levels of anxiety.

**Table 6:** Tukey post hoc test for depression score among significant variables.

Variables		Mean Difference	Std. Error	Sig.
Sleep hours per day				
Less than 4 hours	5–8 hours	4.81676*	1.32525	.001
	9–14 hours	5.53409*	1.69658	.003
Occupation				
Home maid/housekeeping	Security	5.35714*	1.45168	.011
	Driving	3.93516	1.25573	.067
	Food industry	3.91404	1.28054	.083
Working hours				
18–22 hours	8–12 hours	6.51255*	1.47500	.000
	13–17 hours	6.06917*	1.79347	.004
Water intake				
1–3 liters	4–6 liters	1.58102*	.58991	.038
	7–9 liters	3.37943*	1.22111	.030
Anxiety level				
Low	Moderate	-14.97223*	1.18434	.000
	Severe	-23.92678*	3.84865	.000
Country of work	No significant difference			

Table 7 suggests that sleeping hours (F = 3.846, p = 0.022), occupation (F = 2.223, p = 0.016), working hours (F = 6.699, p = 0.000), water intake (F = 3.797, p = 0.010) and anxiety level (F = 14.206, p = 0.00) were found to be significantly associated with the WHO5 wellbeing score.

**Table 7:** Analysis of variance comparisons between WHO 5 wellbeing score with study variables.

Occupation	Mean	Std. Deviation	F	Sig.
Office work	18.5897	4.97741	2.223	.016
Maintenance	18.1429	4.36277		
Aviation	21.2857	2.98408		
Factory workers	18.5417	4.96929		
Food industry	18.5424	4.50028		
Sales	19.6364	4.58134		
Construction	17.7077	4.47464		
Security	19.4857	4.32075		
Driving	18.1538	4.82905		
Garment	19.9167	3.39675		
Home maid/housekeeping	15.9762	5.77838		
**Sleeping hours**				
Less than 4 hours	16.0000	5.36494	3.846	.022
5–8 hours	18.2584	4.64052		
9–14 hours	19.4545	4.84827		
**Working hours**				
3–7 hours	18.0833	4.23102	6.699	.000
8–12 hours	18.3945	4.66046		
13–17 hours	18.7429	4.47439		
18–22 hours	13.5789	4.63460		
**Water intake**				
1–3 Litre	17.6992	4.85630	3.797	.010
4-6Litre	18.5694	4.52198		
7–9 Litre	19.4667	4.43912		
10–12 Litre	22.1429	3.07834		
**BAI level**				
Low	18.4623	4.54856	14.206	.000
Moderate	14.0455	5.02828		
Severe	8.5000	12.02082		

Table 8 depicts the results of the Tukey post hoc test which revealed that the wellbeing score as per the WHO 5 wellbeing scale was significantly lower among respondents with less than 4 hours of sleep compared to those with 9–14 hours of sleep and nearly significantly (P = 0.057) lower than 5–8 hours of sleep. The wellbeing score among security workers was significantly higher than home maids or housekeeping workers. Regarding working hours per day, respondents working for 18–22 hours had a significantly lower wellbeing score compared to those working for 3–7 hours, 8–12 hours, and 13–17 hours. Respondents drinking 7–9 liters of water per day were found to have nearly significantly (P = 0.064) higher wellbeing than those drinking only 1–3 liters of water per day. Furthermore, respondents with a lower level of anxiety had a significantly higher wellbeing score compared to those with moderate and severe anxiety.

**Table 8:** Tukey post hoc test for WHO 5 wellbeing score among significant variables.

Variables		Mean Difference	Std. Error	Sig.
Sleep hours per day				
Less than 4 hours	5–8 hours	-2.25843	.98279	.057
	9–14 hours	-3.45455*	1.25816	.017
Occupation				
Security	Home maid/housekeeping	3.50952*	1.06651	.042
Working hours				
18–22 hours	3–7 hours	-4.50439*	1.71018	.043
	8–12 hours	-4.81555*	1.08696	.000
	13–17 hours	-5.16391*	1.32164	.001
Water intake				
7–9 liters	1–3 liters	4.44364	1.79191	.064
Anxiety level				
Low	Moderate	4.41689*	1.00235	.000
	Severe	9.96234*	3.25724	.007

## Discussion

This study identified the prevalence of anxiety, depression and psychological wellbeing and determined their predictors among Nepalese migrant workers in Gulf countries and Malaysia.

The Nepal Labour Migration Report 2020 stated that majority of migrant workers are male and more than 80% of the total labor migrant population is between the ages of 18 and 35 years with the majority within the age of 25 and 35 years. The trend analysis of labor migration destination countries depicts a large proportion of GCC countries, including the UAE, Qatar, and Kuwait [6]. Our study follows the same trend with 93% of the respondents being male, most respondents belonged to the age group of 26–35 years. Further, most respondents were from Qatar, Saudi Arabia, the UAE and Kuwait. Majority of South Asian migrant workers to Gulf countries are employed in laborious and hazardous jobs [13, 14]. Migrant workers who originated from 25 low-income countries, including Nepal, were found to be involved in unskilled manual labor [29]. Our findings are consistent with these studies.

A systematic review including 36 studies stated that though psychiatric problems were explored in few studies, but this aspect of migrant workers’ health still seems less explored [29]. Psychological issues among these labor migrant workers are least explored, while the current study’s findings depict the prevalence of anxiety and depression among these group.

Previous study conducted among male Nepalese construction workers in Malaysia, Qatar, and Saudi Arabia, found the prevalence of mental health issues among 23% of the migrants [30]. Study among returnee migrants from Qatar, Saudi Arab, Malaysia, Oman, and UAE identified various mental health problems among them as tension, anxiety, and attempts to suicide [31]. These findings support our study where almost 15% of the respondents have mild to severe depression and almost 5% have moderate to severe levels of anxiety.

Moreover, a study from Qatar, including 26% (655) Nepalese migrant workers, concluded that compared with Arabs, Nepalese migrant workers experienced a 4% increase in the predicted probability of depressive symptoms for every unit increase in perceived quality of life [32]. Another study explored the mental health needs of immigrant workers in Gulf countries with the majority of migrants from the Indian sub-continent. Among housemaids, psychiatric morbidity was twice as common as in the local population, and stress-related disorders were the most common (49%) [33]. Though small in number, the current study had female migrant workers who were mainly involved as home-maids and housekeepers. Findings revealed that this group of occupations had significantly higher depression scores and lower wellbeing scores. Similarly, a retrospective analysis of returnee female Nepalese migrant workers from GCC and Malaysia, depicts that 8.3% had mental health problems, 37% had experienced abuse at the workplace, 30.9% had experienced torture or maltreatment at the workplace, 51.7% experienced sexual abuse and 10.9% experienced physical harm [34].

The WHO5 wellbeing index score suggested that in the current study, 14.1% of the respondents had a score below 13, suggestive of poor psychological wellbeing. Previous study conducted to explore migrant worker’s wellbeing and its determinants in Qatar found the wellbeing of Nepalese migrant workers to be 4.793 points lower than the baseline (Indian). A major determinant of migrant wellbeing was contract-related matters. Furthermore, this study identified a low awareness of their legal rights under Gulf labor law [35]. In the current study, wellbeing was found to be affected by work-related variables and anxiety. These studies move in the same direction; work related vulnerabilities can be addressed through clear contract content and proper enforcement, which can provide better wellbeing to migrant workers.

In our study, depression as well as wellbeing were found to be significantly different based on living and work-related variables such as occupation, working hours, sleep hours, water intake and anxiety. Similar results were demonstrated among Asian immigrants in the Middle East; poor living and working conditions attributed to an increased risk of mental illness [11]. This is further enlightened by the report of the International Trade Union Confederation which emphasized the impact of working conditions in Qatar, stating: “Whether the cause of death is labeled a work accident, heart attack or disease from squalid living conditions, the root cause is the same-working condition.” Longer work hours in the heat is risky [36].

A systematic review reported that anxiety-depressive symptoms were more prevalent among migrant workers than in residents. Migrant workers’ personal history (previous physical illness, infrequent visits to hometown), socioeconomic context (e.g., poor living conditions), and work environment (e.g., low monthly income, working in multiple cities, working more than 8 hours per day) all have a significant impact on the development of mental health problems [37]. Similar finding was noted in the context of Nepal as well. Nepali male migrant workers reported a high risk of developing mental health issues compared to non-migrants. Further study suggested that the adverse living and working conditions faced by migrant workers contribute to poor mental and physical health [38]. These findings are congruent with our results.

In current study fewer sleeping hours and lower water intake were found to be significantly associated with higher scores in depression measures and lower scores in wellbeing measures. Similar findings have been reported by some studies. A strong association of sleep deprivation with mental health morbidities (depression, traumatic disorder, and anxiety) has been reported in previous study [39]. Also, a study exploring the relationship between water intake and the risk of depression and anxiety found that the lowest level of water drinking (<2 glasses/day) compared with the reference group (≥5 glasses/day) increased the risk for depression (OR = 1.79; 95% CI: 1.32,2.42; P<0.0001), after adjusting for potential confounders. Water drinking < 2 glasses/day was found to be associated with a 73% and 54% increment in the risk of depression in men and women, respectively (P< 0.05) [40].

Previous study conducted among Nepali migrant workers who returned permanently from six Gulf countries included their family members as well. Results showed that more than half, 56%, of returnees suffered from anxiety, 23% had depression, 6% had suicidal ideation or even suicide attempts, and a further 11% had serious mental illness. This prevalence is greater than in the current study. It might be because of the limited time for data collection (one month), which may not capture all possible scenarios of mental health, and our study included those who returned permanently as well as temporarily. Work-related problems such as arduous work other than described in their contract, low pay, illegal status, and violence (threat, verbal, and physical assaults) were identified as contributing factors to mental health problems. Moreover, 56% of those left behind family member had anxiety, 26% had depression, 7% had suicidal ideation or attempted suicide, and 2% had severe mental illness according to a study. Study found that health issues of migrant workers, the death of migrant workers, and difficulty in communication between migrant workers and family members contributed to mental health problems in family members of migrant workers [41].

Another retrospective analysis of deaths of Nepalese migrant workers revealed that out of 1354 samples, the cause of death was suicide (8.5%) [42]. Another study looked for depression and suicidal behaviors among male migrant workers in the United Arab Emirates and including migrants from South Asian countries. Depression was found to be correlated with physical illness, working in the construction industry, less income, and working more than 8 hours a day. Also, suicidal ideation was higher among those who had physical illness, less income, and worked for more than 8 hours a day [43]. Suicide can result from neglected psychological morbidities such as anxiety and depression.

The findings of our study are important as they represent a vulnerable group that if not addressed timely, might even be linked to severe mental illness and suicide ideations as well as attempts among these groups along with their family members, as captured by the above study findings.

An increasing need for the assessment and understanding of various aspects of migrants’ health along with their mental health has resulted from overarching globalization and urbanization. [44].

For migrants from Indian sub-continent pre-immigration risk factors, lack of contact with family members back home, and harassment at work [36], uncertainty in employment, discrimination, and changes in social status [45] are found to be risk factors for mental health problems. If they develop mental health problems, there prevails various barriers such as language, inadequate access to health care, and social stigma for mental health morbidity, making it hard for them to get the available treatment. This has a direct impact on their wellbeing. Further, if they seek treatment for mental health issues, the nature of migration hinders continuity of treatment [39]. Study among Nepalese returnee migrants from Qatar, Saudi Arab, Malaysia, Oman, and UAE concluded that migrant workers deal with poor access to mental health services, along with poor communication facilities, unfair treatment and discrimination at work, poor working and living arrangements, social isolation, lack of security, loneliness, and poor social life at work. Study also claimed that the predeparture training package holds only formality: contents good but poor implementation [34]. Thus, pre-immigration assessment of psychological history and risk factors, training and orientation related to their job responsibility, language, and culture to familiarize workers is vital [36]. Thus, ensuring quality of pre-immigration assessment and services along with prioritizing migrant workers overall health (physical and mental health) by host and destination countries is warranted.

The result has to be interpreted as per limitation. There is a chance of selection bias in the study; migrants with more psychological problems might not have given consent to participate. Furthermore, there was a time limitation as permission for data collection was obtained only for a month, so data from this point of time is not sufficient to draw conclusions for all the migrant workers. Only a few female migrant workers were included in the study. Bivariate analysis has been used in the study while more advanced analysis such as multivariate analysis could have been performed. The dependent variables were not normally distributed while other assumptions for ANOVA were met. Furthermore, while interpreting the results, it has to be considered that some of the cell sizes were small in comparison to others. The variables were measured with different categories/levels to represent the lowest as well as the highest value for the variable. Also, there is the chance of recall bias as the data collection tools are required to know the response referring to past two weeks. Further, when a migrant worker is planning to return to their home country, in the one or two weeks before the flight, they might be preoccupied with flying back, which can have an impact in their thoughts, feelings, and even behaviors. Thus, the two-week recall period of the instruments in this context has to be considered while interpreting the results.

## Conclusion and recommendation

Our findings suggests that Nepalese migrant workers in GCC countries and Malaysia have psychological morbidities impacting their wellbeing. Living and working conditions are found to be associated with depression and psychological wellbeing of Nepalese migrant workers. This highlights the need for enhanced and comprehensive pre-immigration sessions and screenings. Further psychological screening and counseling for migrant workers with poor working conditions should be prioritized in Gulf countries and Malaysia. This study indicates the need for collaborative effort for mental health of migrant workers by Nepal and Gulf countries and Malaysia.

## Supporting information

S1 File (DOCX)
