# Supplementary material for: Psychological morbidities among Nepalese migrant workers to Gulf and Malaysia
Source: PLoS One. 2023 Nov 8;18(11):e0267784. doi: 10.1371/journal.pone.0267784 (PMC10631658; doi:10.1371/journal.pone.0267784)
Supplement: S1 File — (DOCX) [file pone.0267784.s001.docx]

| **Case Processing Summary** | | | | | | |
| --- | --- | --- | --- | --- | --- | --- |
|  | Cases | | | | | |
|  | Valid | | Missing | | Total | |
|  | N | Percent | N | Percent | N | Percent |
| whototal | 502 | 100.0% | 0 | 0.0% | 502 | 100.0% |

| **Descriptives** | | | | |
| --- | --- | --- | --- | --- |
|  | | | Statistic | Std. Error |
| whototal | Mean | | 18.2291 | .21051 |
|  | 95% Confidence Interval for Mean | Lower Bound | 17.8155 |  |
|  |  | Upper Bound | 18.6427 |  |
|  | 5% Trimmed Mean | | 18.5071 |  |
|  | Median | | 19.0000 |  |
|  | Variance | | 22.245 |  |
|  | Std. Deviation | | 4.71644 |  |
|  | Minimum | | .00 |  |
|  | Maximum | | 25.00 |  |
|  | Range | | 25.00 |  |
|  | Interquartile Range | | 7.00 |  |
|  | Skewness | | -.782 | .109 |
|  | Kurtosis | | .457 | .218 |

| **Tests of Normality** | | | | | | |
| --- | --- | --- | --- | --- | --- | --- |
|  | Kolmogorov-Smirnov^a^ | | | Shapiro-Wilk | | |
|  | Statistic | df | Sig. | Statistic | df | Sig. |
| whototal | .105 | 502 | .000 | .949 | 502 | .000 |
| a. Lilliefors Significance Correction | | | | | | |

| **Case Processing Summary** | | | | | | |
| --- | --- | --- | --- | --- | --- | --- |
|  | Cases | | | | | |
|  | Valid | | Missing | | Total | |
|  | N | Percent | N | Percent | N | Percent |
| BDI_Sum | 502 | 100.0% | 0 | 0.0% | 502 | 100.0% |

| **Descriptives** | | | | |
| --- | --- | --- | --- | --- |
|  | | | Statistic | Std. Error |
| BDI_Sum | Mean | | 4.3247 | .28560 |
|  | 95% Confidence Interval for Mean | Lower Bound | 3.7636 |  |
|  |  | Upper Bound | 4.8858 |  |
|  | 5% Trimmed Mean | | 3.4486 |  |
|  | Median | | 1.0000 |  |
|  | Variance | | 40.946 |  |
|  | Std. Deviation | | 6.39893 |  |
|  | Minimum | | .00 |  |
|  | Maximum | | 35.00 |  |
|  | Range | | 35.00 |  |
|  | Interquartile Range | | 6.00 |  |
|  | Skewness | | 2.022 | .109 |
|  | Kurtosis | | 4.199 | .218 |

| **Tests of Normality** | | | | | | |
| --- | --- | --- | --- | --- | --- | --- |
|  | Kolmogorov-Smirnov^a^ | | | Shapiro-Wilk | | |
|  | Statistic | df | Sig. | Statistic | df | Sig. |
| BDI_Sum | .250 | 502 | .000 | .717 | 502 | .000 |
| a. Lilliefors Significance Correction | | | | | | |

ONEWAY BDI_Sum BY COUNTRY_OF_WORK_EXPERIENCE

/STATISTICS DESCRIPTIVES

/MISSING ANALYSIS

/POSTHOC=TUKEY ALPHA(0.05).

| **Descriptives** | | | | | | | | |
| --- | --- | --- | --- | --- | --- | --- | --- | --- |
| BDI_Sum | | | | | | | | |
|  | N | Mean | Std. Deviation | Std. Error | 95% Confidence Interval for Mean | | Minimum | Maximum |
|  |  |  |  |  | Lower Bound | Upper Bound |  |  |
| Qatar | 210 | 4.4571 | 6.14113 | .42378 | 3.6217 | 5.2926 | .00 | 35.00 |
| Saudi Arabia | 107 | 3.5607 | 5.94975 | .57518 | 2.4204 | 4.7011 | .00 | 30.00 |
| UAE | 104 | 3.4231 | 6.16708 | .60473 | 2.2237 | 4.6224 | .00 | 31.00 |
| Kuwait | 35 | 6.4857 | 7.37791 | 1.24710 | 3.9513 | 9.0201 | .00 | 25.00 |
| Oman | 14 | 8.2857 | 9.98790 | 2.66938 | 2.5189 | 14.0526 | .00 | 29.00 |
| Bahrain | 5 | 2.4000 | 2.30217 | 1.02956 | -.4585 | 5.2585 | .00 | 5.00 |
| Malaysia | 25 | 5.4800 | 7.05998 | 1.41200 | 2.5658 | 8.3942 | .00 | 24.00 |
| Abuabudabi | 2 | 3.0000 | 2.82843 | 2.00000 | -22.4124 | 28.4124 | 1.00 | 5.00 |
| Total | 502 | 4.3247 | 6.39893 | .28560 | 3.7636 | 4.8858 | .00 | 35.00 |

| **ANOVA** | | | | | |
| --- | --- | --- | --- | --- | --- |
| BDI_Sum | | | | | |
|  | Sum of Squares | df | Mean Square | F | Sig. |
| Between Groups | 589.180 | 7 | 84.169 | 2.087 | .043 |
| Within Groups | 19924.894 | 494 | 40.334 |  |  |
| Total | 20514.074 | 501 |  |  |  |

ONEWAY BDI_Sum BY sleepcatagory

/STATISTICS DESCRIPTIVES

/MISSING ANALYSIS

/POSTHOC=TUKEY ALPHA(0.05).

| **Descriptives** | | | | | | | | |
| --- | --- | --- | --- | --- | --- | --- | --- | --- |
| BDI_Sum | | | | | | | | |
|  | N | Mean | Std. Deviation | Std. Error | 95% Confidence Interval for Mean | | Minimum | Maximum |
|  |  |  |  |  | Lower Bound | Upper Bound |  |  |
| 1.00 | 24 | 8.9583 | 8.58957 | 1.75334 | 5.3313 | 12.5854 | .00 | 35.00 |
| 2.00 | 445 | 4.1416 | 6.19177 | .29352 | 3.5647 | 4.7184 | .00 | 32.00 |
| 3.00 | 33 | 3.4242 | 6.22008 | 1.08278 | 1.2187 | 5.6298 | .00 | 29.00 |
| Total | 502 | 4.3247 | 6.39893 | .28560 | 3.7636 | 4.8858 | .00 | 35.00 |

| **ANOVA** | | | | | |
| --- | --- | --- | --- | --- | --- |
| BDI_Sum | | | | | |
|  | Sum of Squares | df | Mean Square | F | Sig. |
| Between Groups | 556.974 | 2 | 278.487 | 6.963 | .001 |
| Within Groups | 19957.100 | 499 | 39.994 |  |  |
| Total | 20514.074 | 501 |  |  |  |

| **Multiple Comparisons** | | | | | | |
| --- | --- | --- | --- | --- | --- | --- |
| Dependent Variable: BDI_Sum  Tukey HSD | | | | | | |
| (I) sleepcatagory | (J) sleepcatagory | Mean Difference (I-J) | Std. Error | Sig. | 95% Confidence Interval | |
|  |  |  |  |  | Lower Bound | Upper Bound |
| 1.00 | 2.00 | 4.81676^*^ | 1.32525 | .001 | 1.7015 | 7.9321 |
|  | 3.00 | 5.53409^*^ | 1.69658 | .003 | 1.5459 | 9.5223 |
| 2.00 | 1.00 | -4.81676^*^ | 1.32525 | .001 | -7.9321 | -1.7015 |
|  | 3.00 | .71733 | 1.14097 | .804 | -1.9648 | 3.3994 |
| 3.00 | 1.00 | -5.53409^*^ | 1.69658 | .003 | -9.5223 | -1.5459 |
|  | 2.00 | -.71733 | 1.14097 | .804 | -3.3994 | 1.9648 |
| *. The mean difference is significant at the 0.05 level. | | | | | | |

ONEWAY BDI_Sum BY newcategoryoccupation

/STATISTICS DESCRIPTIVES

/MISSING ANALYSIS

/POSTHOC=TUKEY ALPHA(0.05).

| **Descriptives** | | | | | | | | |
| --- | --- | --- | --- | --- | --- | --- | --- | --- |
| BDI_Sum | | | | | | | | |
|  | N | Mean | Std. Deviation | Std. Error | 95% Confidence Interval for Mean | | Minimum | Maximum |
|  |  |  |  |  | Lower Bound | Upper Bound |  |  |
| 1.00 | 39 | 4.5897 | 7.82613 | 1.25318 | 2.0528 | 7.1267 | .00 | 32.00 |
| 2.00 | 56 | 4.3929 | 6.46298 | .86365 | 2.6621 | 6.1237 | .00 | 25.00 |
| 3.00 | 7 | 2.0000 | 2.88675 | 1.09109 | -.6698 | 4.6698 | .00 | 8.00 |
| 4.00 | 24 | 5.4167 | 6.39916 | 1.30622 | 2.7145 | 8.1188 | .00 | 24.00 |
| 5.00 | 59 | 3.7288 | 6.33227 | .82439 | 2.0786 | 5.3790 | .00 | 30.00 |
| 6.00 | 33 | 3.4545 | 5.39149 | .93854 | 1.5428 | 5.3663 | .00 | 20.00 |
| 7.00 | 130 | 4.3231 | 6.17976 | .54200 | 3.2507 | 5.3954 | .00 | 29.00 |
| 8.00 | 35 | 2.2857 | 3.97471 | .67185 | .9204 | 3.6511 | .00 | 19.00 |
| 9.00 | 65 | 3.7077 | 6.07691 | .75375 | 2.2019 | 5.2135 | .00 | 35.00 |
| 10.00 | 12 | 5.3333 | 8.29385 | 2.39423 | .0637 | 10.6030 | .00 | 29.00 |
| 11.00 | 42 | 7.6429 | 7.48599 | 1.15511 | 5.3101 | 9.9757 | .00 | 27.00 |
| Total | 502 | 4.3247 | 6.39893 | .28560 | 3.7636 | 4.8858 | .00 | 35.00 |

| **ANOVA** | | | | | |
| --- | --- | --- | --- | --- | --- |
| BDI_Sum | | | | | |
|  | Sum of Squares | df | Mean Square | F | Sig. |
| Between Groups | 760.275 | 10 | 76.028 | 1.890 | .044 |
| Within Groups | 19753.799 | 491 | 40.232 |  |  |
| Total | 20514.074 | 501 |  |  |  |

| **Multiple Comparisons** | | | | | | |
| --- | --- | --- | --- | --- | --- | --- |
| Dependent Variable: BDI_Sum  Tukey HSD | | | | | | |
| (I) newcategoryoccupation | (J) newcategoryoccupation | Mean Difference (I-J) | Std. Error | Sig. | 95% Confidence Interval | |
|  |  |  |  |  | Lower Bound | Upper Bound |
| 1.00 | 2.00 | .19689 | 1.32288 | 1.000 | -4.0813 | 4.4751 |
|  | 3.00 | 2.58974 | 2.60365 | .996 | -5.8304 | 11.0099 |
|  | 4.00 | -.82692 | 1.64557 | 1.000 | -6.1487 | 4.4948 |
|  | 5.00 | .86093 | 1.30900 | 1.000 | -3.3724 | 5.0942 |
|  | 6.00 | 1.13520 | 1.50024 | 1.000 | -3.7166 | 5.9870 |
|  | 7.00 | .26667 | 1.15804 | 1.000 | -3.4784 | 4.0118 |
|  | 8.00 | 2.30403 | 1.47684 | .898 | -2.4721 | 7.0801 |
|  | 9.00 | .88205 | 1.28473 | 1.000 | -3.2727 | 5.0369 |
|  | 10.00 | -.74359 | 2.09386 | 1.000 | -7.5151 | 6.0279 |
|  | 11.00 | -3.05311 | 1.41049 | .531 | -7.6146 | 1.5084 |
| 2.00 | 1.00 | -.19689 | 1.32288 | 1.000 | -4.4751 | 4.0813 |
|  | 3.00 | 2.39286 | 2.54280 | .997 | -5.8305 | 10.6162 |
|  | 4.00 | -1.02381 | 1.54750 | 1.000 | -6.0284 | 3.9808 |
|  | 5.00 | .66404 | 1.18335 | 1.000 | -3.1629 | 4.4910 |
|  | 6.00 | .93831 | 1.39197 | 1.000 | -3.5633 | 5.4399 |
|  | 7.00 | .06978 | 1.01385 | 1.000 | -3.2090 | 3.3486 |
|  | 8.00 | 2.10714 | 1.36671 | .905 | -2.3128 | 6.5271 |
|  | 9.00 | .68516 | 1.15645 | 1.000 | -3.0548 | 4.4251 |
|  | 10.00 | -.94048 | 2.01769 | 1.000 | -7.4657 | 5.5847 |
|  | 11.00 | -3.25000 | 1.29473 | .301 | -7.4371 | .9371 |
| 3.00 | 1.00 | -2.58974 | 2.60365 | .996 | -11.0099 | 5.8304 |
|  | 2.00 | -2.39286 | 2.54280 | .997 | -10.6162 | 5.8305 |
|  | 4.00 | -3.41667 | 2.72465 | .976 | -12.2281 | 5.3948 |
|  | 5.00 | -1.72881 | 2.53560 | 1.000 | -9.9289 | 6.4713 |
|  | 6.00 | -1.45455 | 2.63942 | 1.000 | -9.9904 | 7.0813 |
|  | 7.00 | -2.32308 | 2.46107 | .997 | -10.2821 | 5.6360 |
|  | 8.00 | -.28571 | 2.62619 | 1.000 | -8.7788 | 8.2073 |
|  | 9.00 | -1.70769 | 2.52316 | 1.000 | -9.8676 | 6.4522 |
|  | 10.00 | -3.33333 | 3.01663 | .991 | -13.0891 | 6.4224 |
|  | 11.00 | -5.64286 | 2.58946 | .521 | -14.0171 | 2.7314 |
| 4.00 | 1.00 | .82692 | 1.64557 | 1.000 | -4.4948 | 6.1487 |
|  | 2.00 | 1.02381 | 1.54750 | 1.000 | -3.9808 | 6.0284 |
|  | 3.00 | 3.41667 | 2.72465 | .976 | -5.3948 | 12.2281 |
|  | 5.00 | 1.68785 | 1.53565 | .991 | -3.2784 | 6.6541 |
|  | 6.00 | 1.96212 | 1.70161 | .987 | -3.5409 | 7.4651 |
|  | 7.00 | 1.09359 | 1.40918 | 1.000 | -3.4637 | 5.6509 |
|  | 8.00 | 3.13095 | 1.68101 | .741 | -2.3054 | 8.5673 |
|  | 9.00 | 1.70897 | 1.51502 | .989 | -3.1906 | 6.6085 |
|  | 10.00 | .08333 | 2.24254 | 1.000 | -7.1690 | 7.3357 |
|  | 11.00 | -2.22619 | 1.62303 | .955 | -7.4750 | 3.0227 |
| 5.00 | 1.00 | -.86093 | 1.30900 | 1.000 | -5.0942 | 3.3724 |
|  | 2.00 | -.66404 | 1.18335 | 1.000 | -4.4910 | 3.1629 |
|  | 3.00 | 1.72881 | 2.53560 | 1.000 | -6.4713 | 9.9289 |
|  | 4.00 | -1.68785 | 1.53565 | .991 | -6.6541 | 3.2784 |
|  | 6.00 | .27427 | 1.37878 | 1.000 | -4.1847 | 4.7332 |
|  | 7.00 | -.59426 | .99568 | 1.000 | -3.8143 | 2.6257 |
|  | 8.00 | 1.44310 | 1.35328 | .993 | -2.9334 | 5.8196 |
|  | 9.00 | .02112 | 1.14055 | 1.000 | -3.6674 | 3.7096 |
|  | 10.00 | -1.60452 | 2.00862 | .999 | -8.1004 | 4.8913 |
|  | 11.00 | -3.91404 | 1.28054 | .083 | -8.0553 | .2272 |
| 6.00 | 1.00 | -1.13520 | 1.50024 | 1.000 | -5.9870 | 3.7166 |
|  | 2.00 | -.93831 | 1.39197 | 1.000 | -5.4399 | 3.5633 |
|  | 3.00 | 1.45455 | 2.63942 | 1.000 | -7.0813 | 9.9904 |
|  | 4.00 | -1.96212 | 1.70161 | .987 | -7.4651 | 3.5409 |
|  | 5.00 | -.27427 | 1.37878 | 1.000 | -4.7332 | 4.1847 |
|  | 7.00 | -.86853 | 1.23637 | 1.000 | -4.8669 | 3.1299 |
|  | 8.00 | 1.16883 | 1.53903 | 1.000 | -3.8084 | 6.1460 |
|  | 9.00 | -.25315 | 1.35576 | 1.000 | -4.6377 | 4.1314 |
|  | 10.00 | -1.87879 | 2.13817 | .999 | -8.7936 | 5.0360 |
|  | 11.00 | -4.18831 | 1.47548 | .147 | -8.9600 | .5834 |
| 7.00 | 1.00 | -.26667 | 1.15804 | 1.000 | -4.0118 | 3.4784 |
|  | 2.00 | -.06978 | 1.01385 | 1.000 | -3.3486 | 3.2090 |
|  | 3.00 | 2.32308 | 2.46107 | .997 | -5.6360 | 10.2821 |
|  | 4.00 | -1.09359 | 1.40918 | 1.000 | -5.6509 | 3.4637 |
|  | 5.00 | .59426 | .99568 | 1.000 | -2.6257 | 3.8143 |
|  | 6.00 | .86853 | 1.23637 | 1.000 | -3.1299 | 4.8669 |
|  | 8.00 | 2.03736 | 1.20787 | .842 | -1.8689 | 5.9436 |
|  | 9.00 | .61538 | .96355 | 1.000 | -2.5007 | 3.7315 |
|  | 10.00 | -1.01026 | 1.91367 | 1.000 | -7.1990 | 5.1785 |
|  | 11.00 | -3.31978 | 1.12578 | .111 | -6.9605 | .3210 |
| 8.00 | 1.00 | -2.30403 | 1.47684 | .898 | -7.0801 | 2.4721 |
|  | 2.00 | -2.10714 | 1.36671 | .905 | -6.5271 | 2.3128 |
|  | 3.00 | .28571 | 2.62619 | 1.000 | -8.2073 | 8.7788 |
|  | 4.00 | -3.13095 | 1.68101 | .741 | -8.5673 | 2.3054 |
|  | 5.00 | -1.44310 | 1.35328 | .993 | -5.8196 | 2.9334 |
|  | 6.00 | -1.16883 | 1.53903 | 1.000 | -6.1460 | 3.8084 |
|  | 7.00 | -2.03736 | 1.20787 | .842 | -5.9436 | 1.8689 |
|  | 9.00 | -1.42198 | 1.32982 | .993 | -5.7226 | 2.8786 |
|  | 10.00 | -3.04762 | 2.12182 | .939 | -9.9096 | 3.8143 |
|  | 11.00 | -5.35714^*^ | 1.45168 | .011 | -10.0519 | -.6624 |
| 9.00 | 1.00 | -.88205 | 1.28473 | 1.000 | -5.0369 | 3.2727 |
|  | 2.00 | -.68516 | 1.15645 | 1.000 | -4.4251 | 3.0548 |
|  | 3.00 | 1.70769 | 2.52316 | 1.000 | -6.4522 | 9.8676 |
|  | 4.00 | -1.70897 | 1.51502 | .989 | -6.6085 | 3.1906 |
|  | 5.00 | -.02112 | 1.14055 | 1.000 | -3.7096 | 3.6674 |
|  | 6.00 | .25315 | 1.35576 | 1.000 | -4.1314 | 4.6377 |
|  | 7.00 | -.61538 | .96355 | 1.000 | -3.7315 | 2.5007 |
|  | 8.00 | 1.42198 | 1.32982 | .993 | -2.8786 | 5.7226 |
|  | 10.00 | -1.62564 | 1.99289 | .999 | -8.0706 | 4.8193 |
|  | 11.00 | -3.93516 | 1.25573 | .067 | -7.9962 | .1258 |
| 10.00 | 1.00 | .74359 | 2.09386 | 1.000 | -6.0279 | 7.5151 |
|  | 2.00 | .94048 | 2.01769 | 1.000 | -5.5847 | 7.4657 |
|  | 3.00 | 3.33333 | 3.01663 | .991 | -6.4224 | 13.0891 |
|  | 4.00 | -.08333 | 2.24254 | 1.000 | -7.3357 | 7.1690 |
|  | 5.00 | 1.60452 | 2.00862 | .999 | -4.8913 | 8.1004 |
|  | 6.00 | 1.87879 | 2.13817 | .999 | -5.0360 | 8.7936 |
|  | 7.00 | 1.01026 | 1.91367 | 1.000 | -5.1785 | 7.1990 |
|  | 8.00 | 3.04762 | 2.12182 | .939 | -3.8143 | 9.9096 |
|  | 9.00 | 1.62564 | 1.99289 | .999 | -4.8193 | 8.0706 |
|  | 11.00 | -2.30952 | 2.07619 | .990 | -9.0239 | 4.4048 |
| 11.00 | 1.00 | 3.05311 | 1.41049 | .531 | -1.5084 | 7.6146 |
|  | 2.00 | 3.25000 | 1.29473 | .301 | -.9371 | 7.4371 |
|  | 3.00 | 5.64286 | 2.58946 | .521 | -2.7314 | 14.0171 |
|  | 4.00 | 2.22619 | 1.62303 | .955 | -3.0227 | 7.4750 |
|  | 5.00 | 3.91404 | 1.28054 | .083 | -.2272 | 8.0553 |
|  | 6.00 | 4.18831 | 1.47548 | .147 | -.5834 | 8.9600 |
|  | 7.00 | 3.31978 | 1.12578 | .111 | -.3210 | 6.9605 |
|  | 8.00 | 5.35714^*^ | 1.45168 | .011 | .6624 | 10.0519 |
|  | 9.00 | 3.93516 | 1.25573 | .067 | -.1258 | 7.9962 |
|  | 10.00 | 2.30952 | 2.07619 | .990 | -4.4048 | 9.0239 |
| *. The mean difference is significant at the 0.05 level. | | | | | | |

ONEWAY BDI_Sum BY working_hour_recoded

/STATISTICS DESCRIPTIVES

/MISSING ANALYSIS

/POSTHOC=TUKEY ALPHA(0.05).

| **Descriptives** | | | | | | | | |
| --- | --- | --- | --- | --- | --- | --- | --- | --- |
| BDI_Sum | | | | | | | | |
|  | N | Mean | Std. Deviation | Std. Error | 95% Confidence Interval for Mean | | Minimum | Maximum |
|  |  |  |  |  | Lower Bound | Upper Bound |  |  |
| 3-7 | 12 | 5.4167 | 9.53899 | 2.75367 | -.6441 | 11.4775 | .00 | 29.00 |
| 8-12 | 436 | 4.0138 | 6.07991 | .29117 | 3.4415 | 4.5860 | .00 | 35.00 |
| 13-17 | 35 | 4.4571 | 6.86141 | 1.15979 | 2.1002 | 6.8141 | .00 | 30.00 |
| 18-22 | 19 | 10.5263 | 7.61846 | 1.74779 | 6.8543 | 14.1983 | .00 | 27.00 |
| Total | 502 | 4.3247 | 6.39893 | .28560 | 3.7636 | 4.8858 | .00 | 35.00 |

| **ANOVA** | | | | | |
| --- | --- | --- | --- | --- | --- |
| BDI_Sum | | | | | |
|  | Sum of Squares | df | Mean Square | F | Sig. |
| Between Groups | 787.817 | 3 | 262.606 | 6.630 | .000 |
| Within Groups | 19726.257 | 498 | 39.611 |  |  |
| Total | 20514.074 | 501 |  |  |  |

| **Multiple Comparisons** | | | | | | |
| --- | --- | --- | --- | --- | --- | --- |
| Dependent Variable: BDI_Sum  Tukey HSD | | | | | | |
| (I) working_hour_recoded | (J) working_hour_recoded | Mean Difference (I-J) | Std. Error | Sig. | 95% Confidence Interval | |
|  |  |  |  |  | Lower Bound | Upper Bound |
| 3-7 | 8-12 | 1.40291 | 1.84167 | .872 | -3.3444 | 6.1502 |
|  | 13-17 | .95952 | 2.10539 | .968 | -4.4676 | 6.3866 |
|  | 18-22 | -5.10965 | 2.32071 | .124 | -11.0918 | .8725 |
| 8-12 | 3-7 | -1.40291 | 1.84167 | .872 | -6.1502 | 3.3444 |
|  | 13-17 | -.44338 | 1.10571 | .978 | -3.2936 | 2.4068 |
|  | 18-22 | -6.51255^*^ | 1.47500 | .000 | -10.3147 | -2.7104 |
| 13-17 | 3-7 | -.95952 | 2.10539 | .968 | -6.3866 | 4.4676 |
|  | 8-12 | .44338 | 1.10571 | .978 | -2.4068 | 3.2936 |
|  | 18-22 | -6.06917^*^ | 1.79347 | .004 | -10.6922 | -1.4461 |
| 18-22 | 3-7 | 5.10965 | 2.32071 | .124 | -.8725 | 11.0918 |
|  | 8-12 | 6.51255^*^ | 1.47500 | .000 | 2.7104 | 10.3147 |
|  | 13-17 | 6.06917^*^ | 1.79347 | .004 | 1.4461 | 10.6922 |
| *. The mean difference is significant at the 0.05 level. | | | | | | |

ONEWAY BDI_Sum BY water_intake_amount_perday

/STATISTICS DESCRIPTIVES

/MISSING ANALYSIS

/POSTHOC=TUKEY ALPHA(0.05).

| **Descriptives** | | | | | | | | |
| --- | --- | --- | --- | --- | --- | --- | --- | --- |
| BDI_Sum | | | | | | | | |
|  | N | Mean | Std. Deviation | Std. Error | 95% Confidence Interval for Mean | | Minimum | Maximum |
|  |  |  |  |  | Lower Bound | Upper Bound |  |  |
| 1-3 L | 256 | 5.2461 | 7.08146 | .44259 | 4.3745 | 6.1177 | .00 | 32.00 |
| 4-6L | 209 | 3.6651 | 5.72887 | .39627 | 2.8838 | 4.4463 | .00 | 35.00 |
| 7-9 L | 30 | 1.8667 | 3.19194 | .58277 | .6748 | 3.0586 | .00 | 13.00 |
| 10-12 L | 7 | .8571 | 2.26779 | .85714 | -1.2402 | 2.9545 | .00 | 6.00 |
| Total | 502 | 4.3247 | 6.39893 | .28560 | 3.7636 | 4.8858 | .00 | 35.00 |

| **ANOVA** | | | | | |
| --- | --- | --- | --- | --- | --- |
| BDI_Sum | | | | | |
|  | Sum of Squares | df | Mean Square | F | Sig. |
| Between Groups | 573.699 | 3 | 191.233 | 4.776 | .003 |
| Within Groups | 19940.375 | 498 | 40.041 |  |  |
| Total | 20514.074 | 501 |  |  |  |

| **Multiple Comparisons** | | | | | | |
| --- | --- | --- | --- | --- | --- | --- |
| Dependent Variable: BDI_Sum  Tukey HSD | | | | | | |
| (I) water_intake_amount_perday | (J) water_intake_amount_perday | Mean Difference (I-J) | Std. Error | Sig. | 95% Confidence Interval | |
|  |  |  |  |  | Lower Bound | Upper Bound |
| 1-3 L | 4-6L | 1.58102^*^ | .58991 | .038 | .0604 | 3.1016 |
|  | 7-9 L | 3.37943^*^ | 1.22111 | .030 | .2317 | 6.5271 |
|  | 10-12 L | 4.38895 | 2.42416 | .270 | -1.8599 | 10.6378 |
| 4-6L | 1-3 L | -1.58102^*^ | .58991 | .038 | -3.1016 | -.0604 |
|  | 7-9 L | 1.79841 | 1.23543 | .465 | -1.3862 | 4.9830 |
|  | 10-12 L | 2.80793 | 2.43140 | .656 | -3.4596 | 9.0754 |
| 7-9 L | 1-3 L | -3.37943^*^ | 1.22111 | .030 | -6.5271 | -.2317 |
|  | 4-6L | -1.79841 | 1.23543 | .465 | -4.9830 | 1.3862 |
|  | 10-12 L | 1.00952 | 2.65609 | .981 | -5.8372 | 7.8562 |
| 10-12 L | 1-3 L | -4.38895 | 2.42416 | .270 | -10.6378 | 1.8599 |
|  | 4-6L | -2.80793 | 2.43140 | .656 | -9.0754 | 3.4596 |
|  | 7-9 L | -1.00952 | 2.65609 | .981 | -7.8562 | 5.8372 |
| *. The mean difference is significant at the 0.05 level. | | | | | | |

ONEWAY BDI_Sum BY BAI_level

/STATISTICS DESCRIPTIVES

/MISSING ANALYSIS

/POSTHOC=TUKEY ALPHA(0.05).

| **Descriptives** | | | | | | | | |
| --- | --- | --- | --- | --- | --- | --- | --- | --- |
| BDI_Sum | | | | | | | | |
|  | N | Mean | Std. Deviation | Std. Error | 95% Confidence Interval for Mean | | Minimum | Maximum |
|  |  |  |  |  | Lower Bound | Upper Bound |  |  |
| low | 478 | 3.5732 | 5.39682 | .24684 | 3.0882 | 4.0583 | .00 | 32.00 |
| moderate | 22 | 18.5455 | 5.83689 | 1.24443 | 15.9575 | 21.1334 | 10.00 | 29.00 |
| severe | 2 | 27.5000 | 10.60660 | 7.50000 | -67.7965 | 122.7965 | 20.00 | 35.00 |
| Total | 502 | 4.3247 | 6.39893 | .28560 | 3.7636 | 4.8858 | .00 | 35.00 |

| **ANOVA** | | | | | |
| --- | --- | --- | --- | --- | --- |
| BDI_Sum | | | | | |
|  | Sum of Squares | df | Mean Square | F | Sig. |
| Between Groups | 5793.182 | 2 | 2896.591 | 98.187 | .000 |
| Within Groups | 14720.892 | 499 | 29.501 |  |  |
| Total | 20514.074 | 501 |  |  |  |

| **Multiple Comparisons** | | | | | | |
| --- | --- | --- | --- | --- | --- | --- |
| Dependent Variable: BDI_Sum  Tukey HSD | | | | | | |
| (I) BAI_level | (J) BAI_level | Mean Difference (I-J) | Std. Error | Sig. | 95% Confidence Interval | |
|  |  |  |  |  | Lower Bound | Upper Bound |
| low | moderate | -14.97223^*^ | 1.18434 | .000 | -17.7563 | -12.1882 |
|  | severe | -23.92678^*^ | 3.84865 | .000 | -32.9739 | -14.8797 |
| moderate | low | 14.97223^*^ | 1.18434 | .000 | 12.1882 | 17.7563 |
|  | severe | -8.95455 | 4.01140 | .067 | -18.3843 | .4752 |
| severe | low | 23.92678^*^ | 3.84865 | .000 | 14.8797 | 32.9739 |
|  | moderate | 8.95455 | 4.01140 | .067 | -.4752 | 18.3843 |
| *. The mean difference is significant at the 0.05 level. | | | | | | |

ONEWAY whototal BY sleepcatagory

/STATISTICS DESCRIPTIVES

/MISSING ANALYSIS

/POSTHOC=TUKEY ALPHA(0.05).

| **Descriptives** | | | | | | | | |
| --- | --- | --- | --- | --- | --- | --- | --- | --- |
| whototal | | | | | | | | |
|  | N | Mean | Std. Deviation | Std. Error | 95% Confidence Interval for Mean | | Minimum | Maximum |
|  |  |  |  |  | Lower Bound | Upper Bound |  |  |
| 1.00 | 24 | 16.0000 | 5.36494 | 1.09511 | 13.7346 | 18.2654 | .00 | 25.00 |
| 2.00 | 445 | 18.2584 | 4.64052 | .21998 | 17.8261 | 18.6908 | 3.00 | 25.00 |
| 3.00 | 33 | 19.4545 | 4.84827 | .84397 | 17.7354 | 21.1737 | 7.00 | 25.00 |
| Total | 502 | 18.2291 | 4.71644 | .21051 | 17.8155 | 18.6427 | .00 | 25.00 |

| **ANOVA** | | | | | |
| --- | --- | --- | --- | --- | --- |
| whototal | | | | | |
|  | Sum of Squares | df | Mean Square | F | Sig. |
| Between Groups | 169.193 | 2 | 84.596 | 3.846 | .022 |
| Within Groups | 10975.463 | 499 | 21.995 |  |  |
| Total | 11144.655 | 501 |  |  |  |

| **Multiple Comparisons** | | | | | | |
| --- | --- | --- | --- | --- | --- | --- |
| Dependent Variable: whototal  Tukey HSD | | | | | | |
| (I) sleepcatagory | (J) sleepcatagory | Mean Difference (I-J) | Std. Error | Sig. | 95% Confidence Interval | |
|  |  |  |  |  | Lower Bound | Upper Bound |
| 1.00 | 2.00 | -2.25843 | .98279 | .057 | -4.5687 | .0518 |
|  | 3.00 | -3.45455^*^ | 1.25816 | .017 | -6.4121 | -.4970 |
| 2.00 | 1.00 | 2.25843 | .98279 | .057 | -.0518 | 4.5687 |
|  | 3.00 | -1.19612 | .84613 | .335 | -3.1851 | .7929 |
| 3.00 | 1.00 | 3.45455^*^ | 1.25816 | .017 | .4970 | 6.4121 |
|  | 2.00 | 1.19612 | .84613 | .335 | -.7929 | 3.1851 |
| *. The mean difference is significant at the 0.05 level. | | | | | | |

ONEWAY whototal BY newcategoryoccupation

/STATISTICS DESCRIPTIVES

/MISSING ANALYSIS

/POSTHOC=TUKEY ALPHA(0.05).

| **Descriptives** | | | | | | | | |
| --- | --- | --- | --- | --- | --- | --- | --- | --- |
| whototal | | | | | | | | |
|  | N | Mean | Std. Deviation | Std. Error | 95% Confidence Interval for Mean | | Minimum | Maximum |
|  |  |  |  |  | Lower Bound | Upper Bound |  |  |
| 1.00 | 39 | 18.5897 | 4.97741 | .79702 | 16.9763 | 20.2032 | 5.00 | 25.00 |
| 2.00 | 56 | 18.1429 | 4.36277 | .58300 | 16.9745 | 19.3112 | 5.00 | 25.00 |
| 3.00 | 7 | 21.2857 | 2.98408 | 1.12788 | 18.5259 | 24.0455 | 16.00 | 25.00 |
| 4.00 | 24 | 18.5417 | 4.96929 | 1.01435 | 16.4433 | 20.6400 | 10.00 | 25.00 |
| 5.00 | 59 | 18.5424 | 4.50028 | .58589 | 17.3696 | 19.7152 | 8.00 | 25.00 |
| 6.00 | 33 | 19.6364 | 4.58134 | .79751 | 18.0119 | 21.2608 | 4.00 | 25.00 |
| 7.00 | 130 | 17.7077 | 4.47464 | .39245 | 16.9312 | 18.4842 | 4.00 | 25.00 |
| 8.00 | 35 | 19.4857 | 4.32075 | .73034 | 18.0015 | 20.9699 | 10.00 | 25.00 |
| 9.00 | 65 | 18.1538 | 4.82905 | .59897 | 16.9573 | 19.3504 | .00 | 25.00 |
| 10.00 | 12 | 19.9167 | 3.39675 | .98056 | 17.7585 | 22.0749 | 15.00 | 25.00 |
| 11.00 | 42 | 15.9762 | 5.77838 | .89162 | 14.1755 | 17.7769 | 3.00 | 25.00 |
| Total | 502 | 18.2291 | 4.71644 | .21051 | 17.8155 | 18.6427 | .00 | 25.00 |

| **ANOVA** | | | | | |
| --- | --- | --- | --- | --- | --- |
| whototal | | | | | |
|  | Sum of Squares | df | Mean Square | F | Sig. |
| Between Groups | 482.705 | 10 | 48.271 | 2.223 | .016 |
| Within Groups | 10661.950 | 491 | 21.715 |  |  |
| Total | 11144.655 | 501 |  |  |  |

| **Multiple Comparisons** | | | | | | |
| --- | --- | --- | --- | --- | --- | --- |
| Dependent Variable: whototal  Tukey HSD | | | | | | |
| (I) newcategoryoccupation | (J) newcategoryoccupation | Mean Difference (I-J) | Std. Error | Sig. | 95% Confidence Interval | |
|  |  |  |  |  | Lower Bound | Upper Bound |
| 1.00 | 2.00 | .44689 | .97188 | 1.000 | -2.6962 | 3.5899 |
|  | 3.00 | -2.69597 | 1.91282 | .946 | -8.8820 | 3.4901 |
|  | 4.00 | .04808 | 1.20895 | 1.000 | -3.8617 | 3.9578 |
|  | 5.00 | .04737 | .96168 | 1.000 | -3.0627 | 3.1574 |
|  | 6.00 | -1.04662 | 1.10219 | .997 | -4.6111 | 2.5178 |
|  | 7.00 | .88205 | .85078 | .994 | -1.8694 | 3.6335 |
|  | 8.00 | -.89597 | 1.08499 | .999 | -4.4048 | 2.6129 |
|  | 9.00 | .43590 | .94385 | 1.000 | -2.6165 | 3.4883 |
|  | 10.00 | -1.32692 | 1.53830 | .999 | -6.3017 | 3.6479 |
|  | 11.00 | 2.61355 | 1.03625 | .294 | -.7377 | 5.9648 |
| 2.00 | 1.00 | -.44689 | .97188 | 1.000 | -3.5899 | 2.6962 |
|  | 3.00 | -3.14286 | 1.86812 | .844 | -9.1843 | 2.8986 |
|  | 4.00 | -.39881 | 1.13690 | 1.000 | -4.0755 | 3.2779 |
|  | 5.00 | -.39952 | .86937 | 1.000 | -3.2111 | 2.4120 |
|  | 6.00 | -1.49351 | 1.02264 | .932 | -4.8007 | 1.8137 |
|  | 7.00 | .43516 | .74485 | 1.000 | -1.9737 | 2.8440 |
|  | 8.00 | -1.34286 | 1.00408 | .962 | -4.5901 | 1.9043 |
|  | 9.00 | -.01099 | .84961 | 1.000 | -2.7586 | 2.7366 |
|  | 10.00 | -1.77381 | 1.48234 | .983 | -6.5677 | 3.0201 |
|  | 11.00 | 2.16667 | .95120 | .451 | -.9095 | 5.2428 |
| 3.00 | 1.00 | 2.69597 | 1.91282 | .946 | -3.4901 | 8.8820 |
|  | 2.00 | 3.14286 | 1.86812 | .844 | -2.8986 | 9.1843 |
|  | 4.00 | 2.74405 | 2.00172 | .955 | -3.7295 | 9.2176 |
|  | 5.00 | 2.74334 | 1.86284 | .928 | -3.2810 | 8.7677 |
|  | 6.00 | 1.64935 | 1.93911 | .999 | -4.6217 | 7.9204 |
|  | 7.00 | 3.57802 | 1.80808 | .664 | -2.2693 | 9.4253 |
|  | 8.00 | 1.80000 | 1.92939 | .998 | -4.4396 | 8.0396 |
|  | 9.00 | 3.13187 | 1.85369 | .841 | -2.8630 | 9.1267 |
|  | 10.00 | 1.36905 | 2.21623 | 1.000 | -5.7982 | 8.5363 |
|  | 11.00 | 5.30952 | 1.90240 | .165 | -.8428 | 11.4619 |
| 4.00 | 1.00 | -.04808 | 1.20895 | 1.000 | -3.9578 | 3.8617 |
|  | 2.00 | .39881 | 1.13690 | 1.000 | -3.2779 | 4.0755 |
|  | 3.00 | -2.74405 | 2.00172 | .955 | -9.2176 | 3.7295 |
|  | 5.00 | -.00071 | 1.12820 | 1.000 | -3.6493 | 3.6479 |
|  | 6.00 | -1.09470 | 1.25012 | .999 | -5.1376 | 2.9482 |
|  | 7.00 | .83397 | 1.03529 | .999 | -2.5141 | 4.1821 |
|  | 8.00 | -.94405 | 1.23499 | 1.000 | -4.9380 | 3.0499 |
|  | 9.00 | .38782 | 1.11304 | 1.000 | -3.2117 | 3.9874 |
|  | 10.00 | -1.37500 | 1.64753 | .999 | -6.7031 | 3.9531 |
|  | 11.00 | 2.56548 | 1.19239 | .541 | -1.2907 | 6.4217 |
| 5.00 | 1.00 | -.04737 | .96168 | 1.000 | -3.1574 | 3.0627 |
|  | 2.00 | .39952 | .86937 | 1.000 | -2.4120 | 3.2111 |
|  | 3.00 | -2.74334 | 1.86284 | .928 | -8.7677 | 3.2810 |
|  | 4.00 | .00071 | 1.12820 | 1.000 | -3.6479 | 3.6493 |
|  | 6.00 | -1.09399 | 1.01295 | .992 | -4.3699 | 2.1819 |
|  | 7.00 | .83468 | .73149 | .988 | -1.5310 | 3.2003 |
|  | 8.00 | -.94334 | .99422 | .997 | -4.1586 | 2.2719 |
|  | 9.00 | .38853 | .83793 | 1.000 | -2.3213 | 3.0984 |
|  | 10.00 | -1.37429 | 1.47567 | .998 | -6.1466 | 3.3980 |
|  | 11.00 | 2.56618 | .94078 | .191 | -.4763 | 5.6086 |
| 6.00 | 1.00 | 1.04662 | 1.10219 | .997 | -2.5178 | 4.6111 |
|  | 2.00 | 1.49351 | 1.02264 | .932 | -1.8137 | 4.8007 |
|  | 3.00 | -1.64935 | 1.93911 | .999 | -7.9204 | 4.6217 |
|  | 4.00 | 1.09470 | 1.25012 | .999 | -2.9482 | 5.1376 |
|  | 5.00 | 1.09399 | 1.01295 | .992 | -2.1819 | 4.3699 |
|  | 7.00 | 1.92867 | .90833 | .561 | -1.0088 | 4.8662 |
|  | 8.00 | .15065 | 1.13068 | 1.000 | -3.5060 | 3.8073 |
|  | 9.00 | 1.48252 | .99604 | .923 | -1.7387 | 4.7037 |
|  | 10.00 | -.28030 | 1.57086 | 1.000 | -5.3604 | 4.7998 |
|  | 11.00 | 3.66017^*^ | 1.08399 | .032 | .1546 | 7.1658 |
| 7.00 | 1.00 | -.88205 | .85078 | .994 | -3.6335 | 1.8694 |
|  | 2.00 | -.43516 | .74485 | 1.000 | -2.8440 | 1.9737 |
|  | 3.00 | -3.57802 | 1.80808 | .664 | -9.4253 | 2.2693 |
|  | 4.00 | -.83397 | 1.03529 | .999 | -4.1821 | 2.5141 |
|  | 5.00 | -.83468 | .73149 | .988 | -3.2003 | 1.5310 |
|  | 6.00 | -1.92867 | .90833 | .561 | -4.8662 | 1.0088 |
|  | 8.00 | -1.77802 | .88739 | .647 | -4.6478 | 1.0918 |
|  | 9.00 | -.44615 | .70789 | 1.000 | -2.7355 | 1.8432 |
|  | 10.00 | -2.20897 | 1.40592 | .894 | -6.7557 | 2.3377 |
|  | 11.00 | 1.73150 | .82708 | .583 | -.9432 | 4.4063 |
| 8.00 | 1.00 | .89597 | 1.08499 | .999 | -2.6129 | 4.4048 |
|  | 2.00 | 1.34286 | 1.00408 | .962 | -1.9043 | 4.5901 |
|  | 3.00 | -1.80000 | 1.92939 | .998 | -8.0396 | 4.4396 |
|  | 4.00 | .94405 | 1.23499 | 1.000 | -3.0499 | 4.9380 |
|  | 5.00 | .94334 | .99422 | .997 | -2.2719 | 4.1586 |
|  | 6.00 | -.15065 | 1.13068 | 1.000 | -3.8073 | 3.5060 |
|  | 7.00 | 1.77802 | .88739 | .647 | -1.0918 | 4.6478 |
|  | 9.00 | 1.33187 | .97698 | .957 | -1.8277 | 4.4914 |
|  | 10.00 | -.43095 | 1.55884 | 1.000 | -5.4722 | 4.6103 |
|  | 11.00 | 3.50952^*^ | 1.06651 | .042 | .0605 | 6.9586 |
| 9.00 | 1.00 | -.43590 | .94385 | 1.000 | -3.4883 | 2.6165 |
|  | 2.00 | .01099 | .84961 | 1.000 | -2.7366 | 2.7586 |
|  | 3.00 | -3.13187 | 1.85369 | .841 | -9.1267 | 2.8630 |
|  | 4.00 | -.38782 | 1.11304 | 1.000 | -3.9874 | 3.2117 |
|  | 5.00 | -.38853 | .83793 | 1.000 | -3.0984 | 2.3213 |
|  | 6.00 | -1.48252 | .99604 | .923 | -4.7037 | 1.7387 |
|  | 7.00 | .44615 | .70789 | 1.000 | -1.8432 | 2.7355 |
|  | 8.00 | -1.33187 | .97698 | .957 | -4.4914 | 1.8277 |
|  | 10.00 | -1.76282 | 1.46412 | .982 | -6.4978 | 2.9721 |
|  | 11.00 | 2.17766 | .92255 | .395 | -.8058 | 5.1612 |
| 10.00 | 1.00 | 1.32692 | 1.53830 | .999 | -3.6479 | 6.3017 |
|  | 2.00 | 1.77381 | 1.48234 | .983 | -3.0201 | 6.5677 |
|  | 3.00 | -1.36905 | 2.21623 | 1.000 | -8.5363 | 5.7982 |
|  | 4.00 | 1.37500 | 1.64753 | .999 | -3.9531 | 6.7031 |
|  | 5.00 | 1.37429 | 1.47567 | .998 | -3.3980 | 6.1466 |
|  | 6.00 | .28030 | 1.57086 | 1.000 | -4.7998 | 5.3604 |
|  | 7.00 | 2.20897 | 1.40592 | .894 | -2.3377 | 6.7557 |
|  | 8.00 | .43095 | 1.55884 | 1.000 | -4.6103 | 5.4722 |
|  | 9.00 | 1.76282 | 1.46412 | .982 | -2.9721 | 6.4978 |
|  | 11.00 | 3.94048 | 1.52531 | .260 | -.9924 | 8.8733 |
| 11.00 | 1.00 | -2.61355 | 1.03625 | .294 | -5.9648 | .7377 |
|  | 2.00 | -2.16667 | .95120 | .451 | -5.2428 | .9095 |
|  | 3.00 | -5.30952 | 1.90240 | .165 | -11.4619 | .8428 |
|  | 4.00 | -2.56548 | 1.19239 | .541 | -6.4217 | 1.2907 |
|  | 5.00 | -2.56618 | .94078 | .191 | -5.6086 | .4763 |
|  | 6.00 | -3.66017^*^ | 1.08399 | .032 | -7.1658 | -.1546 |
|  | 7.00 | -1.73150 | .82708 | .583 | -4.4063 | .9432 |
|  | 8.00 | -3.50952^*^ | 1.06651 | .042 | -6.9586 | -.0605 |
|  | 9.00 | -2.17766 | .92255 | .395 | -5.1612 | .8058 |
|  | 10.00 | -3.94048 | 1.52531 | .260 | -8.8733 | .9924 |
| *. The mean difference is significant at the 0.05 level. | | | | | | |

ONEWAY whototal BY working_hour_recoded

/STATISTICS DESCRIPTIVES

/MISSING ANALYSIS

/POSTHOC=TUKEY ALPHA(0.05).

| **Descriptives** | | | | | | | | |
| --- | --- | --- | --- | --- | --- | --- | --- | --- |
| whototal | | | | | | | | |
|  | N | Mean | Std. Deviation | Std. Error | 95% Confidence Interval for Mean | | Minimum | Maximum |
|  |  |  |  |  | Lower Bound | Upper Bound |  |  |
| 3-7 | 12 | 18.0833 | 4.23102 | 1.22139 | 15.3951 | 20.7716 | 11.00 | 25.00 |
| 8-12 | 436 | 18.3945 | 4.66046 | .22320 | 17.9558 | 18.8332 | .00 | 25.00 |
| 13-17 | 35 | 18.7429 | 4.47439 | .75631 | 17.2059 | 20.2799 | 9.00 | 25.00 |
| 18-22 | 19 | 13.5789 | 4.63460 | 1.06325 | 11.3451 | 15.8128 | 3.00 | 22.00 |
| Total | 502 | 18.2291 | 4.71644 | .21051 | 17.8155 | 18.6427 | .00 | 25.00 |

| **ANOVA** | | | | | |
| --- | --- | --- | --- | --- | --- |
| whototal | | | | | |
|  | Sum of Squares | df | Mean Square | F | Sig. |
| Between Groups | 432.275 | 3 | 144.092 | 6.699 | .000 |
| Within Groups | 10712.381 | 498 | 21.511 |  |  |
| Total | 11144.655 | 501 |  |  |  |

| **Multiple Comparisons** | | | | | | |
| --- | --- | --- | --- | --- | --- | --- |
| Dependent Variable: whototal  Tukey HSD | | | | | | |
| (I) working_hour_recoded | (J) working_hour_recoded | Mean Difference (I-J) | Std. Error | Sig. | 95% Confidence Interval | |
|  |  |  |  |  | Lower Bound | Upper Bound |
| 3-7 | 8-12 | -.31116 | 1.35717 | .996 | -3.8096 | 3.1872 |
|  | 13-17 | -.65952 | 1.55150 | .974 | -4.6589 | 3.3398 |
|  | 18-22 | 4.50439^*^ | 1.71018 | .043 | .0960 | 8.9128 |
| 8-12 | 3-7 | .31116 | 1.35717 | .996 | -3.1872 | 3.8096 |
|  | 13-17 | -.34836 | .81482 | .974 | -2.4487 | 1.7520 |
|  | 18-22 | 4.81555^*^ | 1.08696 | .000 | 2.0137 | 7.6174 |
| 13-17 | 3-7 | .65952 | 1.55150 | .974 | -3.3398 | 4.6589 |
|  | 8-12 | .34836 | .81482 | .974 | -1.7520 | 2.4487 |
|  | 18-22 | 5.16391^*^ | 1.32164 | .001 | 1.7571 | 8.5707 |
| 18-22 | 3-7 | -4.50439^*^ | 1.71018 | .043 | -8.9128 | -.0960 |
|  | 8-12 | -4.81555^*^ | 1.08696 | .000 | -7.6174 | -2.0137 |
|  | 13-17 | -5.16391^*^ | 1.32164 | .001 | -8.5707 | -1.7571 |
| *. The mean difference is significant at the 0.05 level. | | | | | | |

ONEWAY whototal BY water_intake_amount_perday

/STATISTICS DESCRIPTIVES

/MISSING ANALYSIS

/POSTHOC=TUKEY ALPHA(0.05).

| **Descriptives** | | | | | | | | |
| --- | --- | --- | --- | --- | --- | --- | --- | --- |
| whototal | | | | | | | | |
|  | N | Mean | Std. Deviation | Std. Error | 95% Confidence Interval for Mean | | Minimum | Maximum |
|  |  |  |  |  | Lower Bound | Upper Bound |  |  |
| 1-3 L | 256 | 17.6992 | 4.85630 | .30352 | 17.1015 | 18.2969 | 3.00 | 25.00 |
| 4-6L | 209 | 18.5694 | 4.52198 | .31279 | 17.9527 | 19.1860 | .00 | 25.00 |
| 7-9 L | 30 | 19.4667 | 4.43912 | .81047 | 17.8091 | 21.1243 | 5.00 | 25.00 |
| 10-12 L | 7 | 22.1429 | 3.07834 | 1.16350 | 19.2959 | 24.9898 | 16.00 | 25.00 |
| Total | 502 | 18.2291 | 4.71644 | .21051 | 17.8155 | 18.6427 | .00 | 25.00 |

| **ANOVA** | | | | | |
| --- | --- | --- | --- | --- | --- |
| whototal | | | | | |
|  | Sum of Squares | df | Mean Square | F | Sig. |
| Between Groups | 249.248 | 3 | 83.083 | 3.797 | .010 |
| Within Groups | 10895.408 | 498 | 21.878 |  |  |
| Total | 11144.655 | 501 |  |  |  |

| **Multiple Comparisons** | | | | | | |
| --- | --- | --- | --- | --- | --- | --- |
| Dependent Variable: whototal  Tukey HSD | | | | | | |
| (I) water_intake_amount_perday | (J) water_intake_amount_perday | Mean Difference (I-J) | Std. Error | Sig. | 95% Confidence Interval | |
|  |  |  |  |  | Lower Bound | Upper Bound |
| 1-3 L | 4-6L | -.87016 | .43605 | .191 | -1.9942 | .2539 |
|  | 7-9 L | -1.76745 | .90263 | .205 | -4.0942 | .5593 |
|  | 10-12 L | -4.44364 | 1.79191 | .064 | -9.0627 | .1754 |
| 4-6L | 1-3 L | .87016 | .43605 | .191 | -.2539 | 1.9942 |
|  | 7-9 L | -.89729 | .91321 | .759 | -3.2513 | 1.4567 |
|  | 10-12 L | -3.57348 | 1.79726 | .194 | -8.2063 | 1.0594 |
| 7-9 L | 1-3 L | 1.76745 | .90263 | .205 | -.5593 | 4.0942 |
|  | 4-6L | .89729 | .91321 | .759 | -1.4567 | 3.2513 |
|  | 10-12 L | -2.67619 | 1.96335 | .523 | -7.7372 | 2.3848 |
| 10-12 L | 1-3 L | 4.44364 | 1.79191 | .064 | -.1754 | 9.0627 |
|  | 4-6L | 3.57348 | 1.79726 | .194 | -1.0594 | 8.2063 |
|  | 7-9 L | 2.67619 | 1.96335 | .523 | -2.3848 | 7.7372 |

ONEWAY whototal BY BAI_level

/STATISTICS DESCRIPTIVES

/MISSING ANALYSIS

/POSTHOC=TUKEY ALPHA(0.05).

| **Descriptives** | | | | | | | | |
| --- | --- | --- | --- | --- | --- | --- | --- | --- |
| whototal | | | | | | | | |
|  | N | Mean | Std. Deviation | Std. Error | 95% Confidence Interval for Mean | | Minimum | Maximum |
|  |  |  |  |  | Lower Bound | Upper Bound |  |  |
| low | 478 | 18.4623 | 4.54856 | .20805 | 18.0535 | 18.8711 | 3.00 | 25.00 |
| moderate | 22 | 14.0455 | 5.02828 | 1.07203 | 11.8160 | 16.2749 | 5.00 | 23.00 |
| severe | 2 | 8.5000 | 12.02082 | 8.50000 | -99.5027 | 116.5027 | .00 | 17.00 |
| Total | 502 | 18.2291 | 4.71644 | .21051 | 17.8155 | 18.6427 | .00 | 25.00 |

| **ANOVA** | | | | | |
| --- | --- | --- | --- | --- | --- |
| whototal | | | | | |
|  | Sum of Squares | df | Mean Square | F | Sig. |
| Between Groups | 600.379 | 2 | 300.189 | 14.206 | .000 |
| Within Groups | 10544.277 | 499 | 21.131 |  |  |
| Total | 11144.655 | 501 |  |  |  |

| **Multiple Comparisons** | | | | | | |
| --- | --- | --- | --- | --- | --- | --- |
| Dependent Variable: whototal  Tukey HSD | | | | | | |
| (I) BAI_level | (J) BAI_level | Mean Difference (I-J) | Std. Error | Sig. | 95% Confidence Interval | |
|  |  |  |  |  | Lower Bound | Upper Bound |
| low | moderate | 4.41689^*^ | 1.00235 | .000 | 2.0606 | 6.7731 |
|  | severe | 9.96234^*^ | 3.25724 | .007 | 2.3055 | 17.6192 |
| moderate | low | -4.41689^*^ | 1.00235 | .000 | -6.7731 | -2.0606 |
|  | severe | 5.54545 | 3.39498 | .233 | -2.4352 | 13.5261 |
| severe | low | -9.96234^*^ | 3.25724 | .007 | -17.6192 | -2.3055 |
|  | moderate | -5.54545 | 3.39498 | .233 | -13.5261 | 2.4352 |
| *. The mean difference is significant at the 0.05 level. | | | | | | |
